# Effect of Buyang Huanwu decoction for the rehabilitation of ischemic stroke patients: a meta-analysis of randomized controlled trials

**DOI:** 10.1186/s12955-021-01728-6

**Published:** 2021-03-09

**Authors:** Li Gao, Zhuoran Xiao, Chunhua Jia, Wei Wang

**Affiliations:** School of Traditional Chinese Medicine, Beijing University of Chinese Medicine, No. 11 North 3rd Ring East Road, Chaoyang District, Beijing, 100029 China

**Keywords:** Buyang Huanwu decoction, Rehabilitation, Ischemic stroke, Traditional Chinese medicine, Meta-analysis

## Abstract

**Purpose:**

Buyang Huanwu decoction (BHD) is a widely used traditional Chinese medicine for the rehabilitation of ischemic stroke patients in China, but its clinical efficacy and safety have not been adequately assessed. In this paper, we conducted a systematic review and meta-analysis to evaluate the efficacy and safety of BHD.

**Methods:**

We searched seven electronic databases from inception to 31 March 2019. The language was limited to Chinese and English. Randomized controlled trials evaluating the efficacy and safety of BHD for the rehabilitation of ischemic stroke patients were included in the meta-analysis. Reviewers independently performed the screening, data extraction, bias assessment, and data analysis. The treatment efficacy was pooled in a meta-analysis using RevMan 5.3 software with a random-effect model. Any disagreement was resolved by discussion among all reviewers. The PRISMA statement was used in the review process.

**Results:**

A total of 11 studies with 1084 patients were included in the meta-analysis. The results suggested that BHD was superior to other treatments in terms of clinical efficacy in symptoms and daily activities (n = 684, RR = 1.12, 95% CI: 0.99 to 1.27), clinical efficacy in TCM symptoms (n = 280, RR = 1.45, 95% CI: 1.03 to 2.03), National Institute of Health stroke scale (n = 192, MD = 1.66, 95% CI: -1.08 to 4.40), and activities of daily living (n = 200, MD = 8.20, 95% CI: -3.95 to 20.35).

**Conclusions:**

The results supported the clinical use of BHD for the rehabilitation of ischemic stroke patients. However, the methodological qualities of the included studies were relatively low, and there were limited reports on adverse events. The clinical efficacy and safety of BHD need to be further confirmed by more well-designed and high-quality randomized controlled trials to warrant the clinical recommendation of BHD for the rehabilitation of ischemic stroke patients.

**Supplementary Information:**

The online version contains supplementary material available at 10.1186/s12955-021-01728-6.

## Introduction

Ischemic stroke, accounting for approximately 80% of all strokes, is one of the major causes of disability [1, 2]. Even when stroke symptoms are stabilized, stroke survivors’ daily lives are still seriously affected [3]. The goal of stroke rehabilitation is to help patients restore physical and mental functions, and relearn the necessary skills to live everyday life.

There are several approaches to help ischemic stroke patients in rehabilitation, including physical activities (e.g., mobility training [4] and constraint-induced therapy [5]), cognitive and emotional activities (e.g., speech therapy [6]), biological therapies (e.g. stem cells [7]), and some alternative medicines (e.g. acupuncture [8], massage [9], and herbal medicines [10]). In China, traditional Chinese medicine (TCM) is commonly utilized by physicians as a complementary and alternative therapy for rehabilitation.

The commonly used TCM include Buchang Naoxintong, Shuxuetong, and Buyang Huanwu decoction (BHD). Many researchers have reported the therapeutic effect of TCM on ischemic stroke rehabilitation. Xu et al. [11] quantified 16 constituents of Buchang Naoxintong and verified the therapeutic effects of the constituents in ischemic stroke in mice. Han et al. [10] conducted a network meta-analysis to assess the effect of TCM on the recovery of stroke patients and found that Shuxuetong was effective, as it may reduce inflammation and inhibit thrombosis. Mu et al. [12] reported the neuroprotective effects of BHD on cerebral ischemia-induced neuronal damage. They concluded that BHD has a therapeutic effect primarily on facilitating blood circulation, attenuating the inflammatory response, and inhibiting neuronal apoptosis.

BHD was first created by Qingren Wang in the book *Correction of Errors in Medical Classics* (Yi Lin Gai Cuo). This decoction consists of seven herbs: milkvetch root (Huangqi), Chinese angelica (Danggui), red peony root (Chishao), earthworm (Dilong), Szechwan lovage rhizome (Chuanxiong), safflower (Honghua), and peach seed (Taoren). As reported, the main components of these seven herbs have therapeutic effects in promoting the recovery of ischemic stroke. Astragalus polysaccharide, a component of milkvetch roots, has anti-inflammatory effects [13] and resists oxidative stress [14]. Chinese angelica and its component ferulic acid ameliorate nerve injuries caused by cerebral ischemia [15] and augment angiogenesis [16]. Considering the combined effects of multiple herbs in BHD, Wang et al. [17] found that BHD had a neuroprotective effect in rats by decreasing apoptotic cells. Pan et al. [18] studied the mechanism of BHD on neuronal plasticity in cerebral ischemic rats. They found that BHD could facilitate the recovery of the nervous system by improving synaptic plasticity.

The therapeutic effects of BHD on stroke have been systematically reviewed by many researchers. Hao et al. [19] analyzed 19 randomized controlled trials for the clinical efficacy and safety of BHD and provided suggestive evidence for BHD in patients with acute ischemic stroke. Gou et al. [20] conducted a meta-analysis to assess the clinical efficacy of BHD combined with acupuncture in the treatment of stroke. The results showed that BHD could improve the daily activities of patients, and the clinical efficacy of BHD combined with acupuncture is better than that of BHD alone. Han et al. [21] conducted a systematic review and meta-analysis to assess herbal medicine treatments in patients with acute ischemic stroke and concluded that BHD was relatively safe and could improve neurological function. However, none of the systematic reviews or meta-analyses focused on the clinical efficacy and safety of BHD in the rehabilitation stage of ischemic stroke. Therefore, in this study, a meta-analysis was conducted to evaluate the clinical efficacy and safety of BHD on the rehabilitation of ischemic stroke patients.

## Methods

The protocol of this study was registered in PROSPERO with the registration number CRD42018101968.

### Databases and search strategies

We searched several electronic databases from inception to 31 March 2019: the Cochrane Library, Web of Science, PubMed, Chinese Biomedical Literature Database, Chinese National Knowledge Infrastructure, Chinese Scientific Journal Database, and Wan-fang Database. The language was limited to Chinese and English. Search terms were: (Buyang Huanwu decoction OR Buyang Huanwu tang OR Buyang Huanwu formula) AND (stroke OR cerebral infarction OR cerebral embolism OR cerebrovascular accident OR brain attack OR brain ischemia OR apoplexy OR brain vascular accident) AND (randomized controlled trial). The detailed search strategy used in PubMed is supplied in Additional file 1. Additional eligible studies were identified by manual searching relevant systematic reviews and meta-analysis.

### Inclusion criteria

Only randomized controlled trials (RCTs) were included in the meta-analysis.

#### Participants

Participants were diagnosed with ischemic stroke by physical examinations, computed tomography scans, and magnetic resonance imaging scans. The included patients should be in the recovery stage and not in the acute stage. We only included patients with a course of disease of more than two weeks. The patients’ symptoms were medically stable, and the main symptoms were hemiplegia, apraxia, pain, and depression.

#### Interventions

Interventions using BHD or modified BHD alone were included. TCM physicians prescribed a modified BHD according to the patient’s clinical symptoms. The modified BHD has similarities in the major components and primary therapeutic effects compared with BHD. Interventions using combined therapies, including acupuncture, moxibustion, TCM medicines, and western medicines, were excluded.

#### Comparators

The control group should use a therapy other than BHD. Acupuncture, TCM medicines, or Western medicines were all accepted, but not the BHD with some modifications.

#### Outcomes

The primary outcome was clinical efficacy, and additional outcomes included neurological examination, activities of daily living (ADLs), and adverse events. Neurological examination was commonly conducted by using the National Institute of Health Stroke Scale (NIHSS) to measure the patient’s neurological impairment.

### Exclusion criteria

In addition to some exclusion criteria described above, studies were excluded if they were non-RCTs, unpublished or duplicate studies. Studies that reported patients with the following conditions were also excluded: (a) hemorrhagic stroke; (b) multiple recurrent strokes or in the acute stage; (c) diagnosed with severe liver, kidney, digestive, or mental diseases, malignant tumors, or malignant infectious diseases; and (d) allergies to herbal medicines. Because some studies lacked data for the disease course, it was hard to determine if the patients in those studies were in the acute stage. We contacted the corresponding author by Email for the specific information, and the studies without a response in two weeks were excluded.

### Data extraction and quality assessment

Two reviewers (Gao and Xiao) independently selected relevant studies according to the inclusion and exclusion criteria, and any disagreement was resolved by consulting other reviewers (Jia and Wang). Four reviewers (Gao, Xiao, Jia, and Wang) independently extracted the data and assessed the qualities of the included studies. The risk of bias was classified into three categories (low, unclear, or high risk of bias) and assessed using seven criteria recommended by the Cochrane risk of bias tool [22]. Two reviewers (Gao and Jia) independently utilized RevMan 5.3 software to analyze the data. The dichotomous data was pooled and estimated using relative risk (RR) with 95% confidence intervals (CI). The continuous data was pooled and estimated using mean difference (MD) with 95% CI. The I^2^ statistic was used to evaluate the heterogeneity of the pooled data. A random-effects model was used if high heterogeneity (I^2^ > 50%) was observed in the overall meta-analytic estimate. Sensitivity analysis was performed to check the stability of the results by removing each study at one time in a series of meta-analyses. Subgroup analysis was conducted between different treatment durations, different dosage of milkvetch root, and different treatments in the control groups to investigate the potential source of heterogeneity. A p-value of 0.05 or lower in the data analysis was considered to be statistically significant. A funnel plot and Egger’s test were used to assess the publication bias. All reviewers participated in a discussion together to solve any disagreement in the reviewing process.

## Results

### Description of the included studies

**Fig. 1:**
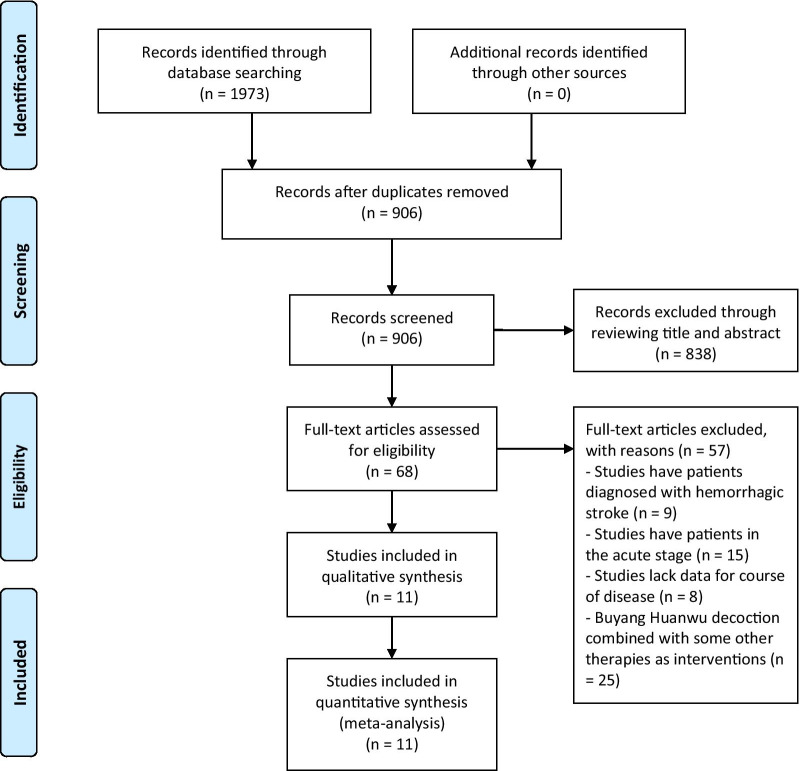
Flowchart of the selection process

This meta-analysis identified 1973 potentially eligible studies (48 in English and 1925 in Chinese). A total of 906 studies were screened by reading the titles and abstracts after removing duplicate publications. A total of 838 studies were excluded because they failed to meet the inclusion criteria. Sixty-eight studies were screened by reading the full texts. We excluded 57 studies, including 9 studies that had patients diagnosed with hemorrhagic stroke, 15 studies that had patients in the acute stage, 8 studies that lacked data for the disease course, and 25 studies that combined BHD with other therapies. Finally, 11 studies [23–33] with 1084 patients were included in the meta-analysis. Detailed information on the selection process is shown in Fig. 1.

Table 1 shows the characteristics of the 11 included studies, which were all written in Chinese. The average age of the patients was greater than 50 years old, and the shortest course of disease was two weeks. There were 554 patients in the experimental group and 530 patients in the control group. In the experimental group, BHD or a modified BHD alone was used for stroke recovery (the details of the interventions are shown in Additional file 2). In the control group, 4 studies [24, 26, 32, 33] used TCM or Chinese patent medicines (CPM), and 7 studies [23, 25, 27–31] used conventional Western medicines. The treatment duration lasted from 4 weeks to 6 months, including 4 studies [23, 25, 28, 31] lasting approximately one month, 1 study [33] lasting two months, 5 studies [24, 26, 27, 29, 30] lasting three months, and 1 study [32] lasting six months. The primary outcomes included clinical efficacy, NIHSS, ADLs, and adverse events. Almost all the studies reported clinical efficacy as an outcome, while only two studies [24, 32] reported NIHSS, two studies [27, 32] reported ADLs, and four studies [26, 27, 31, 32] reported adverse events.

**Table 1:** Characteristics of the 11 included studies

Study	Age (years)	Course of disease	Sample size	(Male/Female)	Intervention group	Control group	Treatment duration
Chang L (2014)	60–70	1–4 y	200 (100/100)	NR	BHD	Piracetam tablet (0.8 g tid)	30 d
Cui H (2016)	(63.8 ± 3.4) (62.4 ± 2.3)	(1.7 ± 0.5) m (2.2 ± 0.6) m	72 (36/36)	(25/11) (23/13)	BHD	Naotong decoction (TCM)	3 m
Jian S (2006)	(58.48 ± 9.06) (56.33 ± 9.13)	18–161 d 22–154 d	76 (40/36)	(23/17) (16/20)	BHD	Vinpocetine (5 mg tid)	4 w
Li S (2013)	(58.64 ± 4.97) (59.87 ± 4.54)	(6.38 ± 1.28) m (6.96 ± 1.59) m	40 (20/20)	(11/9) (13/7)	BHD	Maixuekang capsule (CPM, 0.5 g tid)	3 m
Li S (2016)	(54.6 ± 13.4) (53.7 ± 12.5)	(5.01 ± 3.96) y (4.96 ± 4.01) y	80 (40/40)	(17/23) (20/20)	BHD	Vitamin E (200 mg tid), Aspirin (100 mg qd), Nimodipine (30 mg tid), Venoruton tablet (20 mg tid)	3 m
Luo K (2016)	(65.1 ± 2.9) (65.0 ± 3.0)	(0.6 ± 0.2) y (0.8 ± 0.2) y	80 (40/40)	(21/19) (22/18)	BHD	Conventional western medicine treatment, Nutritional metabolism therapy	30 d
Xu S (2015)	(62.2 ± 1.1) (63.1 ± 1.2)	(6.4 ± 0.4) m (6.1 ± 0.2) m	60 (30/30)	(16/14) (15/15)	BHD	Nimodipine, Vitamin E, Venoruton tablet	3 m
Yan Y (2015)	(60.5 ± 4.8) (61.2 ± 4.5)	(5.6 ± 1.2) m (6.2 ± 1.3) m	82 (41/41)	(25/16) (23/18)	BHD	Aspirin (100 mg qd), Nimodipine (30 mg tid), Venoruton tablet (20 mg bid)	3 m
Ying W (2016)	(54.10 ± 4.7) (54.42 ± 5.6)	16 d—4 m 20 d—5 m	194 (97/97)	(51/46) (62/35)	BHD	Aspirin (100 mg qd), Clopidogrel (75 mg qd)	30 d
Zhang D (2013)	(58.20 ± 10.13) (59.41 ± 9.84)	(4.58 ± 5.29) y (4.79 ± 5.15) y	120 (60/60)	(29/31) (32/28)	BHD	Naoxintong capsule (CPM, 4 capsules tid)	6 m
Zhang H (2018)	(63.65 ± 5.35) (63.55 ± 5.77)	2 w—6 m	80 (30/50)	(16/14) (33/17)	BHD	Peiyuan Tongnao capsule (CPM, 3 capsules tid)	2 m

NR: not reported; BHD: Buyang Huanwu decoction or modified Buyang Huanwu decoction; CPM: Chinese patent medicine; CE: clinical efficacy; NIHSS: National Institute of Health stroke scale; ADLs: activities of daily living; AE: adverse events

### Risk of bias

**Fig. 2:**
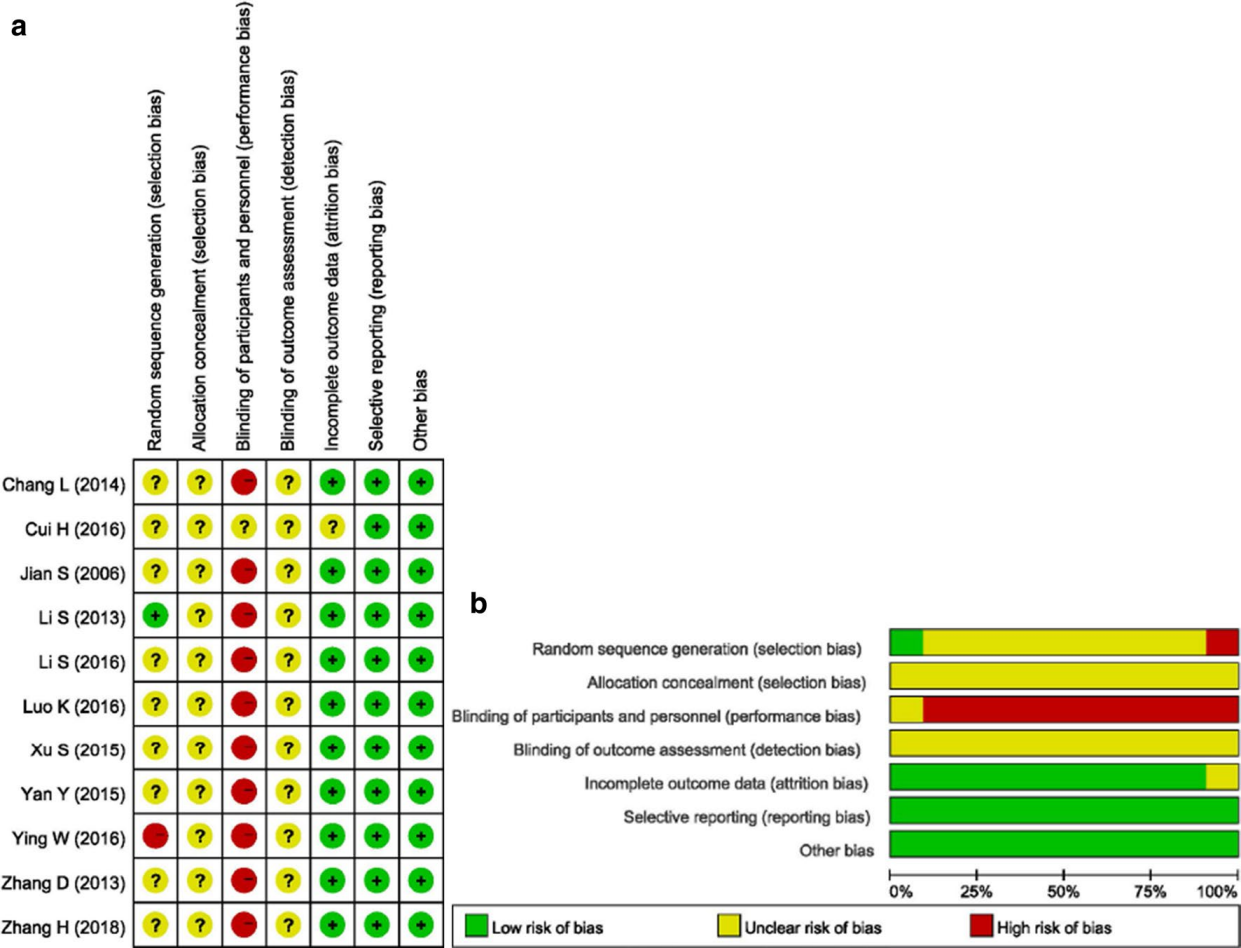
Risk of bias graph

The risk of bias of the included studies is presented in Fig. 2. For selection bias, all the studies reported using a randomization method to generate the sequence; however, most did not report the details. Only one study [26] utilized the drawing of lots, and one study [31] utilized the clinic record number. Due to the characteristics of TCM decoctions, all the participants knew what kind of treatment they had received, making the risk of performance bias high. None of the studies reported the details in selection bias and detection bias, making the biases unclear to be assessed. One study was assessed as unclear risk of bias in incomplete outcome data as the numbers randomized into each intervention group were not clearly reported, while the other studies were still assessed as low risk of bias as all patients completed the study. There was a low risk of bias in selective reporting. In summary, the assessment results suggested that the overall risk of bias was relatively high.

### Outcome measurements

#### Clinical efficacy

Clinical efficiency was utilized as the primary outcome by 10 of the 11 included studies. Eight studies defined clinical efficiency according to the patients’ symptoms and daily activities, while the other two studies defined clinical efficiency based on the patients’ TCM symptoms. The criterion for clinical efficiency in symptoms and daily activities was defined as follows: improvement (symptoms disappeared, activity function fully recovered, and muscle strength recovered, partially recovered or slightly improved) or no effect (symptoms, activity function, and muscle strength were not improved or were even aggravated). The criterion for clinical efficiency in TCM symptoms was defined as follows: improvement (the TCM clinical symptoms were improved, and the syndrome points were reduced by more than 30%) or no effect (the TCM clinical symptoms were not improved or were even aggravated, and the syndrome points were reduced by less than 30%).

**Fig. 3:**
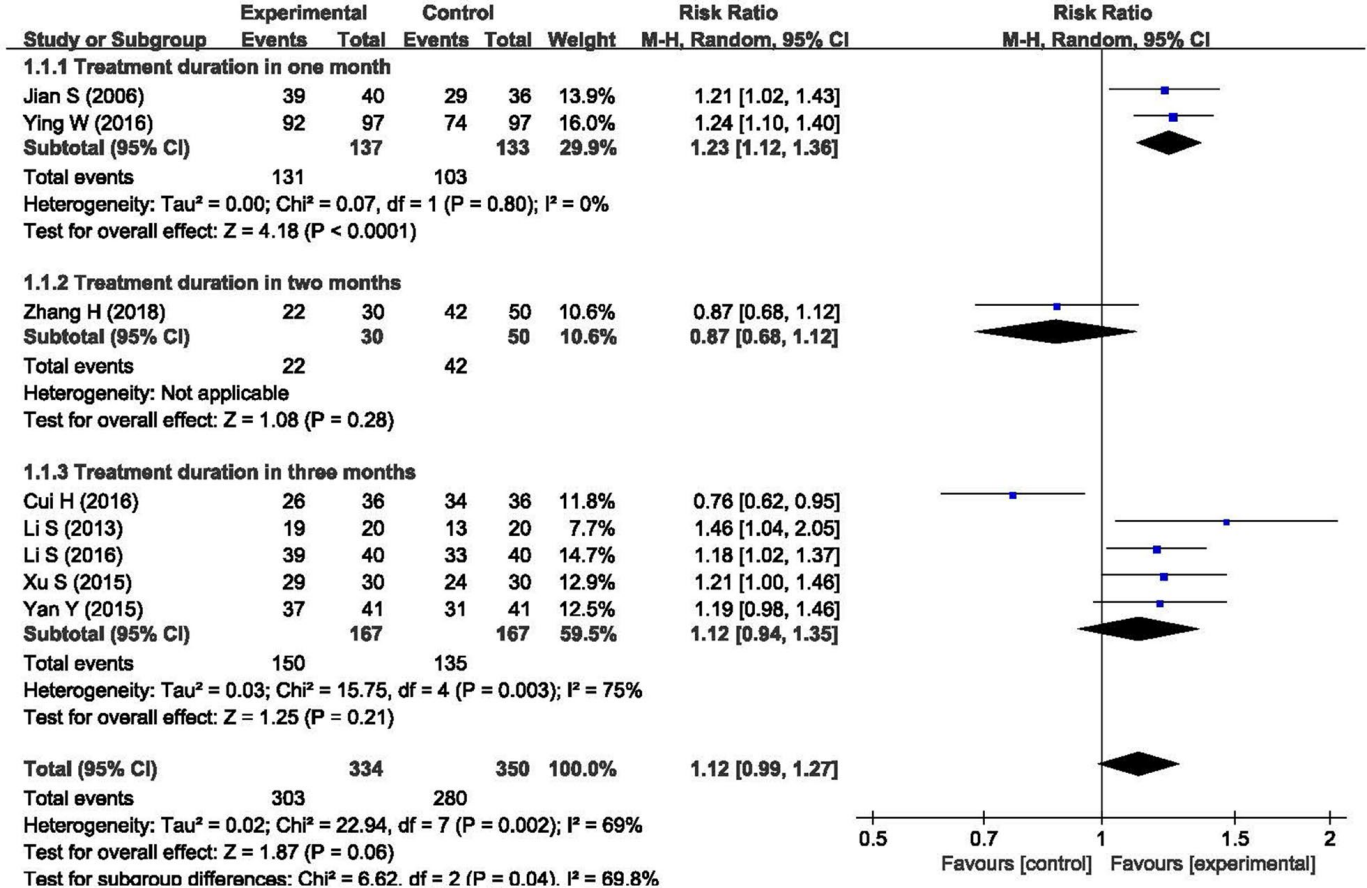
Forest plot of the clinical efficacy in symptoms and daily activities

Figure 3 compares the efficacy in symptoms and daily activities between BHD and other treatments, and the results showed that BHD was effective in ischemic stroke rehabilitation (n = 684, RR = 1.12, 95% CI: 0.99 to 1.27); however, the p-value was 0.06, indicating that the result was not statistically significant. The I^2^ statistic was 69% in the meta-analysis, indicating that the heterogeneity was high. We conducted a subgroup analysis between different treatment durations. The heterogeneity was low in the subgroup of one month (n = 270, RR = 1.23, P < 0.01, I^2^ = 0%), while it was high in the subgroup of three month (n = 334, RR = 1.12, P = 0.21, I^2^ = 75%). The reason for the high heterogeneity in the subgroup of three months was that the study of Cui et al. [24] favored the control group. If we removed this study from the meta-analysis, we found that the subgroup heterogeneity decreased from I^2^ = 75% to I^2^ = 0% (n = 262, RR = 1.21, 95% CI: 1.10 to 1.34, P < 0.01) and the total heterogeneity decreased to I^2^ = 26% (n = 612, RR = 1.19, 95% CI: 1.10 to 1.29, P < 0.01), indicating that the heterogeneity was low.

**Fig. 4:**
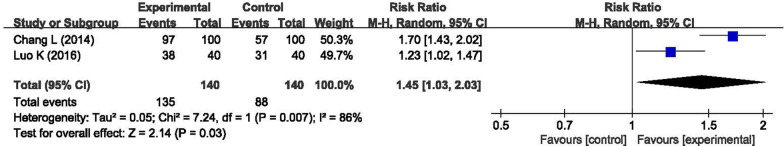
Forest plot of the clinical efficacy in TCM symptoms

Figure 4 compares the efficacy in TCM symptoms between BHD and other treatments, and the results showed that BHD was effective in ischemic stroke rehabilitation (n = 280, RR = 1.45, 95% CI: 1.03 to 2.03, P = 0.03). The heterogeneity was high, with I^2^ = 86%.

#### NIHSS

**Fig. 5:**
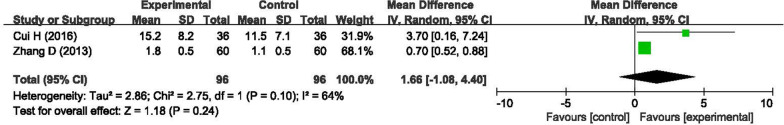
Forest plot of NIHSS in ischemic stroke rehabilitation

Two studies [24, 32] utilized NIHSS to measure patients’ neurological impairment. The forest plot of NIHSS compares the efficacy between BHD and other treatments, and the results showed that BHD was effective in ischemic stroke rehabilitation (n = 192, MD = 1.66, 95% CI: -1.08 to 4.40, P = 0.24), as shown in Fig. 5. The heterogeneity was high, with I^2^ = 64%.

#### ADLs

**Fig. 6:**
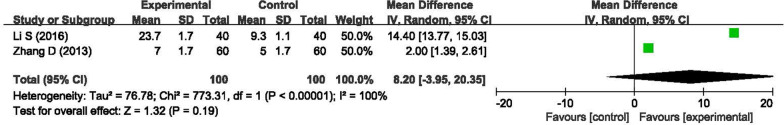
Forest plot of the ADLs in ischemic stroke rehabilitation

Two studies [27, 32] utilized ADLs to assess patients’ activities of daily living. The forest plot of ADLs compares BHD and other treatments’ efficacy, and the results also favored BHD in ischemic stroke rehabilitation (n = 200, MD = 8.20, 95% CI: -3.95 to 20.35, P < 0.01), as shown in Fig. 6. The heterogeneity was high, with I^2^ = 100%.

#### Adverse events (AEs)

Only four studies [26, 27, 31, 32] reported adverse events. Three studies [26, 31, 32] reported no AEs in the experimental or control group. In one study, Li [27] reported that there were no AEs in the experimental group. In contrast, the control group had two cases of abnormal routine blood tests, three cases of high blood sugar levels, one case of blood lipid levels outside the normal range, one case of liver dysfunction, and one case of renal dysfunction. The rate of AEs in the control group was 8/40. The other studies did not report AEs.

## Discussion

In TCM, BHD has been utilized by many physicians for the rehabilitation of ischemic stroke patients. It has been reported that there were several benefits in using this prescription in TCM theory. However, the clinical efficacy of BHD has not been systematically reported to address this controversy. Therefore, the goal of this meta-analysis was to assess the effects and safety of BHD for the rehabilitation of ischemic stroke patients.

A total of 11 studies with 1084 patients with a comparison between BHD and other treatments for the rehabilitation of ischemic stroke patients were included in this meta-analysis. The results of the meta-analysis suggested that BHD was superior to other treatments in terms of clinical efficacy in symptoms and daily activities (n = 684, RR = 1.12, 95% CI: 0.99 to 1.27), clinical efficacy in TCM symptoms (n = 280, RR = 1.45, 95% CI: 1.03 to 2.03), NIHSS (n = 192, MD = 1.66, 95% CI: -1.08 to 4.40), and ADLs (n = 200, MD = 8.20, 95% CI: -3.95 to 20.35).

**Fig. 7:**
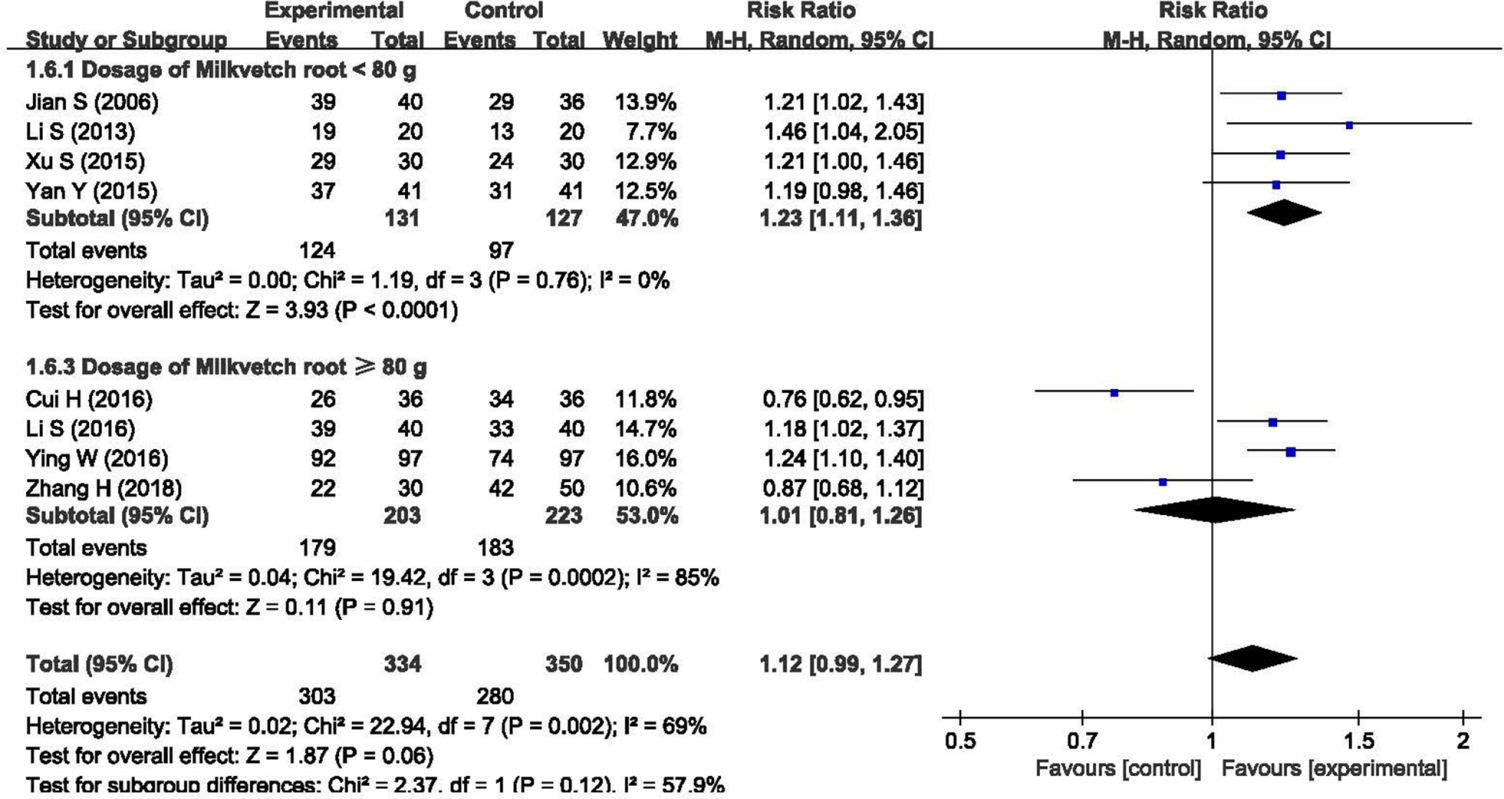
Subgroup analysis between the different dosages of milkvetch root

There was high heterogeneity in this meta-analysis for clinical efficacy in symptoms and daily activities (I^2^ = 69%), clinical efficacy in TCM symptoms (I^2^ = 86%), NIHSS (I^2^ = 64%), and ADLs (I^2^ = 100%). There were four possible reasons for the high heterogeneity. First, a modified BHD was used instead of the original BHD in the treatment, and the diversity of the modified BHD made it challenging to maintain consistency regarding clinical efficacy. In the BHD and the modified BHD, the most important component is the herb milkvetch root. The milkvetch root can replenish Qi in Chinese medicine theory and is also known for reducing oxidative stress and inhibiting the inflammatory response in the treatment mechanism. In addition, the dosage of milkvetch root is significantly higher than that of other herbs, usually 5–10 times the dosage. We conducted a subgroup analysis between the different dosages of milkvetch root, as shown in Fig. 7. The results showed that the subgroup with a dosage of milkvetch root < 80 g had better clinical efficacy (n = 258, RR = 1.23, 95% CI: 1.13 to 1.30) and lower heterogeneity (I^2^ = 0%) than the subgroup with a dosage of milkvetch root ≥ 80 g. A lower dosage of milkvetch root could be better for the rehabilitation of ischemic stroke patients than the commonly recommended dosage of milkvetch root of 120 g.

**Fig. 8:**
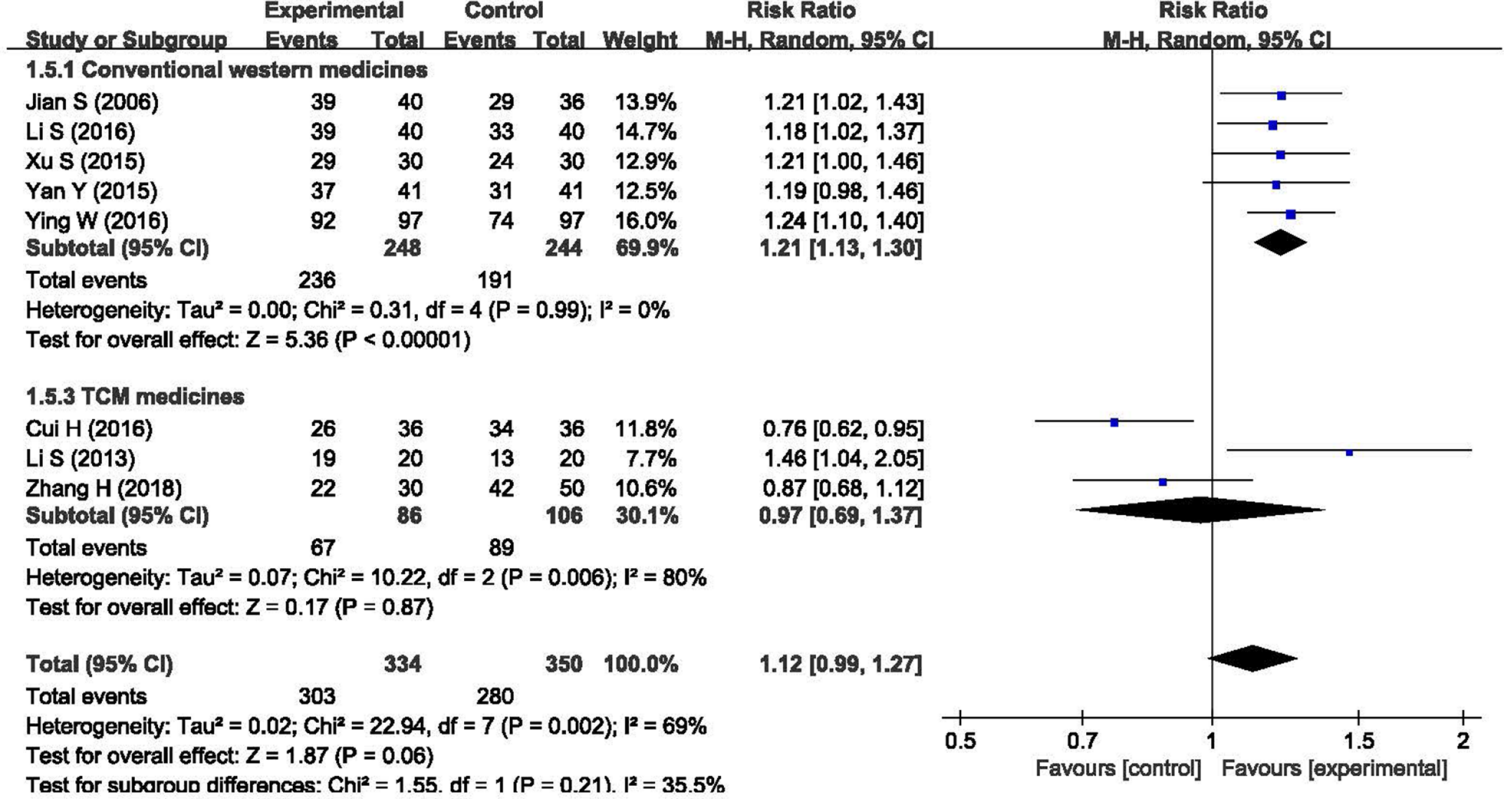
Subgroup analysis between different treatments in the control groups

Second, different treatments were used in different control groups. In the meta-analysis of clinical efficacy in symptoms and daily activities, we conducted another subgroup analysis between different treatments, as shown in Fig. 8. The results showed that there was low heterogeneity in the subgroup of conventional Western medicines (n = 492, RR = 1.21, 95% CI: 1.13 to 1.30), with I^2^ = 0%, while there was high heterogeneity in the subgroup of TCM medicines (n = 192, RR = 0.97, 95% CI: 0.69 to 1.37), with I^2^ = 80%. This finding indicated that the differences in the treatments in the control group could be another reason for the high heterogeneity. In addition, the overall p-value was 0.06, which was mainly attributed to the subgroup of TCM medicines, with a p-value of 0.87.

Third, there were only a small number of patients in the group of clinical efficacy in TCM symptoms, NIHSS, and ADLs, which could easily affect the meta-analysis findings. Fourth, the course of disease in some included studies was as long as five years. As neurological functions are much more difficult to improve at this late stage, the course of disease may be one reason for the high heterogeneity. However, there was not enough data to conduct a subgroup analysis between different courses of disease.

Sensitivity analysis was conducted by removing each study at one time in a series of meta-analyses, as shown in Additional file 3. The results showed that the overall risk ratio was affected most by the study of Cui H (2016) [24]. After removing this study, the overall risk ratio became 1.19, 95% CI: 1.10 to 1.29 (p-value < 0.0001). The meta-analysis remained stable after removing any other study.

**Fig. 9:**
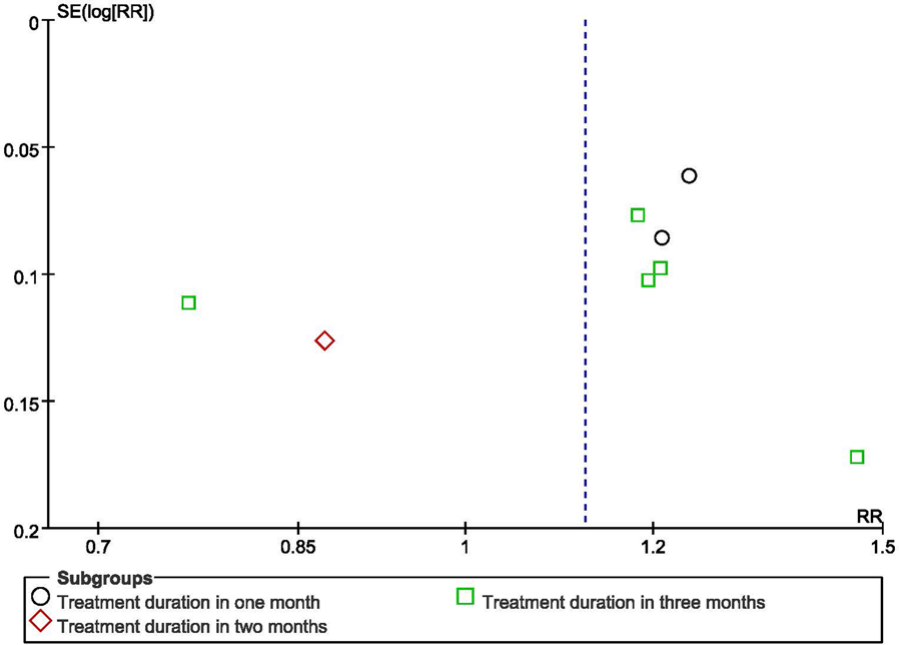
Funnel plot of the clinical efficacy in symptoms and daily activities

In terms of the clinical efficacy in symptoms and daily activities, a funnel plot and Egger’s test were used to assess the publication bias. The Egger regression result was shown in Table 2, and the funnel plot was shown in Fig. 9. The results showed that there was a possible publication bias in the included studies. The main reason was the limited number of included RCT studies and the small sample size of most studies. The potential publication bias may suppress those studies showing a negative result, resulting in an overestimate of the effect of BHD on the rehabilitation of ischemic stroke patients.

**Table 2:** Egger regression to assess publication bias

	Estimate	Standard error	95% CI	p-value
Intercept	− 0.72	4.22	− 10.70 to 9.27	0.87
Slope	0.24	0.74	− 1.50 to 1.98

The methodological qualities of the included studies were relatively low because of the high risk of bias. There were several limitations to this meta-analysis. First, none of the included studies described the details of allocation concealment and blinding, and only two studies reported the randomization methods. Second, most of the included studies had a sample size smaller than 100 patients, which could have caused high heterogeneity in the study. Third, the primary outcomes of the included studies focused on clinical efficacy, and only two studies were assessed by the NIHSS or ADLs scores. The bias in the assessment of clinical efficacy may affect the findings of the meta-analysis. Fourth, limited adverse events of BHD were reported in the included studies, making the safety of BHD hard to be assessed. Fifth, all the included studies were carried out in China and published in Chinese journals. This may make the findings difficult to generalize to other countries.

## Conclusion

In conclusion, this meta-analysis included 11 studies that used BHD or a modified BHD for the rehabilitation of ischemic stroke patients. The results supported the clinical use of BHD in terms of clinical efficacy, NIHSS, ADLs, and safety. However, the methodological qualities of the included studies were relatively low. The safety of BHD could not be clearly assessed because there was limited information on adverse events in the included studies. The clinical efficacy and safety of BHD need to be further confirmed by more well-designed and high-quality RCTs to warrant the clinical recommendation of BHD for the rehabilitation of ischemic stroke patients.

## Supplementary Information

**Additional file 1:** Detailed search strategy used in the PubMed in the metaanalysis.

**Additional file 2:** Composition of formula of the 11 included studies.

**Additional file 3:** Sensitivity analysis in terms of clinical efficacy in symptoms anddaily activities.

## Data Availability

All data generated or analyzed during this study are included in this published article and its additional files.

## Abbreviations

BHD: Buyang Huanwu decoction
TCM: Traditional Chinese medicine
RCTs: Randomized controlled trials
CPM: Chinese patent medicines
NIHSS: National Institute of Health Stroke Scale
ADLs: Activities of daily living
AEs: Adverse events
RR: Relative risk
CI: Confidence intervals
MD: Mean difference
