# Supplementary material for: Effect of Buyang Huanwu decoction for the rehabilitation of ischemic stroke patients: a meta-analysis of randomized controlled trials
Source: Health Qual Life Outcomes. 2021 Mar 9;19:79. doi: 10.1186/s12955-021-01728-6 (PMC7942008; doi:10.1186/s12955-021-01728-6)
Supplement: Supplementary file 1 — Additional file 1: Detailed search strategy used in the PubMed in the metaanalysis. [file 12955_2021_1728_MOESM1_ESM.docx]

Additional file 1 Detailed search strategy used in the PubMed in the meta-analysis

Search ((((((Buyang Huanwu tang) OR Buyang Huanwu formula) OR Buyang Huanwu decoction) OR Buyang Huanwu[MeSH Terms])) AND (((((((Stroke) OR Cerebrovascular accident) OR Brain attack) OR Brain ischemia) OR Apoplexy) OR Brain Vascular Accident) OR Stroke[MeSH Terms])) AND ((randomized controlled trial) OR randomized controlled trial[MeSH Terms])
