# Supplementary material for: Effect of Buyang Huanwu decoction for the rehabilitation of ischemic stroke patients: a meta-analysis of randomized controlled trials
Source: Health Qual Life Outcomes. 2021 Mar 9;19:79. doi: 10.1186/s12955-021-01728-6 (PMC7942008; doi:10.1186/s12955-021-01728-6)
Supplement: Supplementary file 2 — Additional file 2: Composition of formula of the 11 included studies. [file 12955_2021_1728_MOESM2_ESM.docx]

Additional file 2 Composition of formula of the 11 included studies

| Study | Composition of formula |
| --- | --- |
| Chang L (2014) | Milkvetch root 100 g, Chinese angelica 20 g, Red peony root 10 g, Earthworm 10 g, Szechwan lovage rhizome 5 g, Safflower 10 g, Danshen root 10 g, Leech 10 g, Bile arisaema 5 g, Radix puerariae 20 g, Plantain seed 10 g, SanQi 10 g. |
| Cui H (2016) | Milkvetch root 80 g, Chinese angelica 20 g, Red peony root 15 g, Earthworm 10 g, Szechwan lovage rhizome 15 g, Safflower 15 g, Peach seed 15 g, Radix dipsaci 15 g, Twotoothed achyranthes root 15 g. |
| Jian S (2006) | Milkvetch root 60 g, Chinese angelica 10 g, Red peony root 10 g, Earthworm 10 g, Szechwan lovage rhizome 10 g, Safflower 10 g, Peach seed 10 g, Black-tail snake 10 g, Scorpion 5 g. |
| Li S (2013) | Milkvetch root 50 g, Chinese angelica 15 g, Earthworm 15 g, Szechwan lovage rhizome 12 g, Safflower 12 g, Danshen root 25 g, Peach seed 12 g, Beautiful sweetgum fruit 12 g, Hirsute shiny bugleweed herb 12 g. |
| Li S (2016) | Milkvetch root 120 g, Chinese angelica 15 g, Red peony root 10 g, Earthworm 10 g, Szechwan lovage rhizome 10 g, Safflower 10 g, Peach seed 10 g, Liquorice root 10 g, Suberect spatholobus stem 15 g, Twotoothed achyranthes root 20 g, Danshen root 15 g. |
| Luo K (2016) | Milkvetch root 30 g, Chinese angelica 15 g, Red peony root 10 g, Earthworm 10 g, Szechwan lovage rhizome 10 g, Peach seed 10 g, Twotoothed achyranthes root 15 g, Danshen root 15 g. |
| Xu S (2015) | Milkvetch root 60 g, Chinese angelica 10 g, Red peony root 15 g, Safflower 10 g, Peach seed 10 g, Pubescent holly root 10 g, Shinyleaf pricklyash root 15 g, Red tangerine peel 10 g. |
| Yan Y (2015) | Milkvetch root 70 g, Chinese angelica 12 g, Red peony root 12 g, Earthworm 8 g, Szechwan lovage rhizome 10 g, Safflower 12 g, Peach seed 8 g, SanQi 2 g. |
| Ying W (2016) | Milkvetch root 80 g, Chinese angelica 10 g, Red peony root 15 g, Earthworm 10 g, Szechwan lovage rhizome 20 g, Safflower 10 g, Peach seed 10 g, Grassleaf sweetflag rhizome 10 g, Liquorice root 10 g, Twotoothed achyranthes root 15 g, Cassia twig 10 g, Tall gastrodia tuber 10 g, Scorpion 6 g. |
| Zhang D (2013) | Milkvetch root 90 g, Chinese angelica 12 g, Red peony root 10 g, Earthworm 10 g, Szechwan lovage rhizome 9 g, Safflower 10 g, Leech 5 g, Scorpion 3 g. |
| Zhang H (2018) | Milkvetch root 120 g, Chinese angelica 6 g, Red peony root 5 g, Earthworm 3 g, Szechwan lovage rhizome 3 g, Safflower 3 g, Peach seed 3 g. |
