# Supplementary material for: Effect of Buyang Huanwu decoction for the rehabilitation of ischemic stroke patients: a meta-analysis of randomized controlled trials
Source: Health Qual Life Outcomes. 2021 Mar 9;19:79. doi: 10.1186/s12955-021-01728-6 (PMC7942008; doi:10.1186/s12955-021-01728-6)
Supplement: Supplementary file 3 — Additional file 3: Sensitivity analysis in terms of clinical efficacy in symptoms anddaily activities. [file 12955_2021_1728_MOESM3_ESM.docx]

Additional file 3 Sensitivity analysis in terms of clinical efficacy in symptoms and daily activities

| Study Omitted | n | Risk ratio | 95%CI | p-value | I^2^ (%) |
| --- | --- | --- | --- | --- | --- |
| Cui H (2016) | 612 | 1.19 | 1.10-1.29 | < 0.0001 | 26 |
| Jian S (2006) | 608 | 1.11 | 0.96-1.28 | 0.15 | 73 |
| Li S (2013) | 644 | 1.10 | 0.97-1.25 | 0.15 | 72 |
| Li S (2016) | 604 | 1.11 | 0.96-1.28 | 0.15 | 74 |
| Xu S (2015) | 624 | 1.11 | 0.97-1.27 | 0.14 | 73 |
| Yan Y (2015) | 602 | 1.11 | 0.97-1.28 | 0.13 | 74 |
| Ying W (2016) | 490 | 1.10 | 0.95-1.27 | 0.19 | 71 |
| Zhang H (2018) | 604 | 1.16 | 1.03-1.30 | 0.02 | 66 |
